# Meibomian gland dropout rate as a method to assess meibomian gland morphologic changes during use of preservative-containing or preservative-free topical prostaglandin analogues

**DOI:** 10.1371/journal.pone.0218886

**Published:** 2019-06-26

**Authors:** Sang Yeop Lee, Kwanghyun Lee, Chan Keum Park, Sangah Kim, Hyoung Won Bae, Gong Je Seong, Chan Yun Kim

**Affiliations:** 1 Department of Ophthalmology, Severance Hospital, Institute of Vision Research, Yonsei University College of Medicine, Seoul, Korea; 2 Department of Ophthalmology, Maryknoll Hospital, Busan, Korea; 3 Department of Ophthalmology, National Health Insurance Service Ilsan Hospital, Goyang, Korea; Bascom Palmer Eye Institute, UNITED STATES

## Abstract

**Purpose:**

To investigate the usefulness of meibomian gland (MG) dropout rate in the evaluation of MG morphological change associated with the use of prostaglandin for glaucoma treatment through the association between MG and the ocular surface parameters and medication duration and presence of preservative.

**Methods:**

This cross-sectional study was conducted on 88 eyes of 88 patients who were diagnosed with glaucoma and used only Tafluprost as treatment. The patients were divided into four “user” groups: 1) 23 patients used preservative-free (PF) Tafluprost for 6 months; 2) 21 patients used preservative-containing (PC) Tafluprost for 6 months; 3) 23 patients used PF-Tafluprost for 24 months; 4) 21 patients used PC-Tafluprost for 24 months. Ocular surface parameters and the MG condition, including MG dropout rate and meiboscale, were evaluated. Multiple regression was used to identify associations.

**Results:**

There were significant differences in age (p = 0.003), tear breakup time (p = 0.016), lid margin abnormality (p = 0.016), expressibility (p = 0.039), meiboscale (p<0.001), and MG dropout rate (p<0.001) among the 4 groups. MG dropout rate and meiboscale showed significant differences in all post hoc analyses, except for the comparison between the PF-Tafluprost and PC-Tafluprost 6-month user groups. Medication duration, preservative status, and meiboscale were significantly correlated with MG dropout rate (p<0.001, p = 0.024, p<0.001, respectively). In the 6-month user group, preservative status significantly correlated with MG dropout rate (p = 0.015). However, in the 24-month user group, meiboscale was the only parameter significantly associated with MG dropout rate (p<0.001).

**Conclusion:**

MG dropout rate in patients using Tafluprost showed a significant correlation with medication duration and preservative status. This result indicates MG dropout rate reflects MG morphologic change associated with prostaglandin.

## Introduction

Glaucoma is an irreversible progressive disorder of the optic nerve, which can cause blindness if not properly treated. Treatment of glaucoma comprises reduction of intraocular pressure (IOP), which can be achieved with eye drops or laser or surgical methods; however, the primary treatment method is the use of anti-glaucoma eye drops. Continuous use of topical anti-glaucoma medications is necessary to maintain the IOP-lowering effect. A notable limitation in this approach is that long-term use of topical anti-glaucoma medications increases the risk of developing ocular surface diseases [1–5]. Several previous studies have shown that the high incidence of dry eye, as well as the deterioration of ocular parameters and meibomian gland dysfunction (MGD) in glaucoma patients using IOP-lowering eyedrops is associated with the use of topical anti-glaucoma medications [6–10]. Previously, we also reported that glaucoma patients using topical anti-glaucoma medications showed deterioration of ocular surface status, including reduction in the thickness of the tear lipid layer [11].

Meibomian gland (MG) condition in glaucoma patients is related to the behaviors involved in medication use, such as frequency and duration, as well as the type of preservative and active ingredient in the medication [6,7,9,10,12]. Among several active ingredients used as topical anti-glaucoma medications, prostaglandin analogues (PGAs) are known to be associated with development of MGD [8,9,13–15]. Because MGD worsens the condition of the ocular surface [16], an indicator is needed to objectively evaluate MG status in PGA users.

MG dropout rate is an objective parameter that reflects morphologic changes of the MG, indicating loss of the gland. Unlike hyperkeratinization of the MG duct, MG dropout is more closely associated with MG atrophy, suggesting underlying MGD [17,18]. Several previous studies have shown the usefulness of the MG dropout rate for evaluating MGD associated with cataract surgery or periocular radiotherapy [19,20].

Therefore, we conducted this study to evaluate the usefulness of the MG dropout rate for assessing MG status in glaucoma patients using topical PGAs. We analyzed the correlation between medication factors including medication duration and presence or absence of preservative and parameters for ocular surface and MG status, including MG dropout rate. Among topical PGAs, we only used preservative-containing (PC) and preservative-free (PF) Tafluprost (Taflotan and Taflotan-S, Santen Pharmaceutical Co. Ltd., Osaka, Japan) to minimize the effect of medication differences.

## Materials and methods

This cross-sectional study was conducted in the Department of Ophthalmology, Severance Hospital, Yonsei University School of Medicine (Seoul, Korea), and followed the tenets of the Declaration of Helsinki. Ethical approval was obtained from the Institutional Review Board of Severance Hospital, Yonsei University College of Medicine, Seoul, Korea; written informed consent was obtained from all subjects who enrolled in the study. By retrospectively reviewing medical records from the period between January 2015 and December 2017, we identified 88 patients who met the inclusion criteria. The patients were diagnosed with glaucoma for the first time in our glaucoma clinic and began using topical PC- or PF-Tafluprost, which has been used as one of standard treatment methods to treat glaucoma patients in our institution. To the included patients who had no change or addition of topical anti-glaucoma medications during the follow-up period, ophthalmic examinations were performed cross-sectionally at the 6- or 24-month follow-up visit. Patients with a history of ocular surgery, ocular injury, or disease affecting the ocular surface and MG such as allergic conjunctivitis or demodex on eyelashes, as well as patients using other topical agents (e.g., topical steroids, non-steroidal anti-inflammatory drugs, or artificial tears) were excluded in the process of reviewing the medical records of patients. This was confirmed again directly with the patients who visited the outpatient clinic at 6 or 24 months after topical Tafluprost use. In addition, ocular surface and lid margin status were also evaluated at the time of visiting the outpatient clinic to exclude patients having conditions affecting the ocular surface and MG. Among the included patients, we evaluated the ocular surface and MG status in patients who used the medication for 6 months and those who used the medication for 24 months. Included patients did not have abnormal findings regarding the MG or ocular surface conditions in the medical records of their first visit.

Forty-four eyes of 44 glaucoma patients who used topical Tafluprost for 6 months and 44 eyes of 44 glaucoma patients who used topical Tafluprost for 24 months were included in the present study. In each group, 23 eyes of 23 patients used PF-Tafluprost and the remaining 21 eyes of 21 patients used PC-Tafluprost.

As a control group, we enrolled 64 eyes of 64 subjects who visited our hospital for regular check-up or preoperative examination associated with refractive surgery or cataract surgery by reviewing medical records retrospectively. For these subjects, the same examinations for ocular surface and MG conditions were conducted.

### Evaluation of MG and ocular surface status

Ocular surface and MG conditions were evaluated using the same methods as described in previous studies published by ophthalmologists from our institution [11,19,21]. Tear lipid layer thickness (LLT) and MG images of the lower lid (to assess MG dropout rate) were acquired using the LipiView II system (Tear Science, Morrisville, NY, USA). The measurement unit used for LLT was interferometric color units (ICUs), where 1 ICU represented 1 nm of LLT. Among maximum ICU, minimum ICU, and average ICU, average ICU was used. The MG dropout rate in the lower lid was calculated using a previously described method [19]. By automatic threshold identification in the ImageJ software (US National Institutes of Health, Bethesda, MD, USA), the numbers of pixels indicative of total MG area and MG structure area were calculated. Dropout rate was calculated using the following formula: MG dropout rate = [1- (MG structure area / Total MG area)]. Morphologic change of MG was also evaluated using the meiboscale. As previously described, meiboscale is another method for assessing the morphologic change with a score from 0 to 4 points where each point represents a 25% reduction in MG area (0: no reduction, 1: 25% reduction, 2: 50% reduction, 3: 75% reduction, and 4: 100% reduction).

Through direct observation using slit-lamp microscopy, assessments of lid margin abnormality, meibum quality, and meibum expressibility were conducted. Lid margin abnormality was scored from 0 to 4 on the basis of four factors: vascular engorgement, plugging of the MG orifice, anterior or posterior displacement of the mucocutaneous junction, and irregularity of the lid margin. Eight lower lid glands were assessed using a scale of 0 to 3 points at each gland for meibum quality (score range from 0 to 24; grade 0: clear, grade 1: cloudy, grade 2: cloudy with granular debris, and grade 3: thick, like toothpaste). Expressibility of MG was scored by applying digital pressure on five glands of the central one-third of the lower lid, with a score range from 0 to 3 (0: all five glands expressible, 1: three to four glands expressible, 2: one to two glands expressible, 3: no glands expressible).

Tear break up time (TBUT) using a fluorescein strip, type I Schirmer test, and ocular surface staining score (Oxford score) were used to assess ocular surface condition. The ocular surface disease index (OSDI) was used to evaluate subjective ocular discomfort. As described previously [19], the examinations were conducted in the order of LLT measurement and acquiring MG images of the lower lid using the LipiView II system, TBUT, Oxford score, lid margin abnormality, meibum quality, meibum expressibility, OSDI, and Schirmer test to minimize the influence of each examination on the other assessments. All examinations were conducted by two experienced examiners (K.L. and S.K.). Of two eyes in each patient, we included the eye in which the subject complained of greater discomfort. If the degree of discomfort was similar in both eyes, the right eye was selected for inclusion in the present study.

### Statistical analysis

For comparison of continuous and categorical parameters between the groups, the independent two-sample t-test, analyses of variance, and chi-squared tests were performed. A multivariate regression analysis was used to investigate correlations among parameters showing significant differences in group comparisons, including age, sex, medication duration, and presence or absence of preservative in the medication (preservative status). In addition, a multivariate regression analysis was used to identify factors correlated with MG dropout rate on the basis of medication duration and preservative status. All statistical analyses were performed using SAS version 9.4 software (SAS Institute Inc., Cary, NC, USA). Statistical significance was defined as p<0.05.

## Results

Table 1 shows the results of comparisons between non-glaucoma subjects and glaucoma patients using Tafluprost. There were no significant differences in age and sex ratio between the two groups. All parameters for ocular surface and MG condition showed significant differences, except meiboscale.

**Table 1:** Comparison of clinical variables and parameters indicative of ocular surface and meibomian gland conditions between control and glaucoma patients using Tafluprost.

	Control (N = 64) (Mean ± SD or Ratio)	Glaucoma using Tafluprost (N = 88) (Mean ± SD or Ratio)	p*
Age (years)	52.91 ± 20.95	50.77 ± 14.67	0.461
Sex (M:F)	29:35	45:43	0.478
OD:OS	43:21	54:34	0.461
LLT (ICU)	82.50 ± 20.39	68.88 ± 24.19	<0.001
TBUT (s)	6.70 ± 3.78	4.95 ± 2.98	0.002
Dye staining	0.29 ± 0.39	1.17 ± 0.95	<0.001
Schirmer (mm)	12.09 ± 7.97	9.14 ± 5.05	0.005
OSDI	9.86 ± 9.27	26.68 ± 19.96	<0.001
Lid margin abnormality	1.17 ± 1.	1.67 ± 1.15	0.006
Meibum quality	6.03 ± 4.89	9.84 ± 5.92	<0.001
Expressibility	0.58 ± 0.69	1.05 ± 0.91	0.001
Meiboscale	1.19 ± 0.91	1.39 ± 0.95	0.197
MG dropout rate	0.396 ± 0.08	0.434 ± 0.11	0.023

* Independent t-test or chi-squared test. LLT, lipid layer thickness; TBUT, tear breakup time; OSDI, ocular surface disease index; MG, meibomian gland

**Fig 1:**
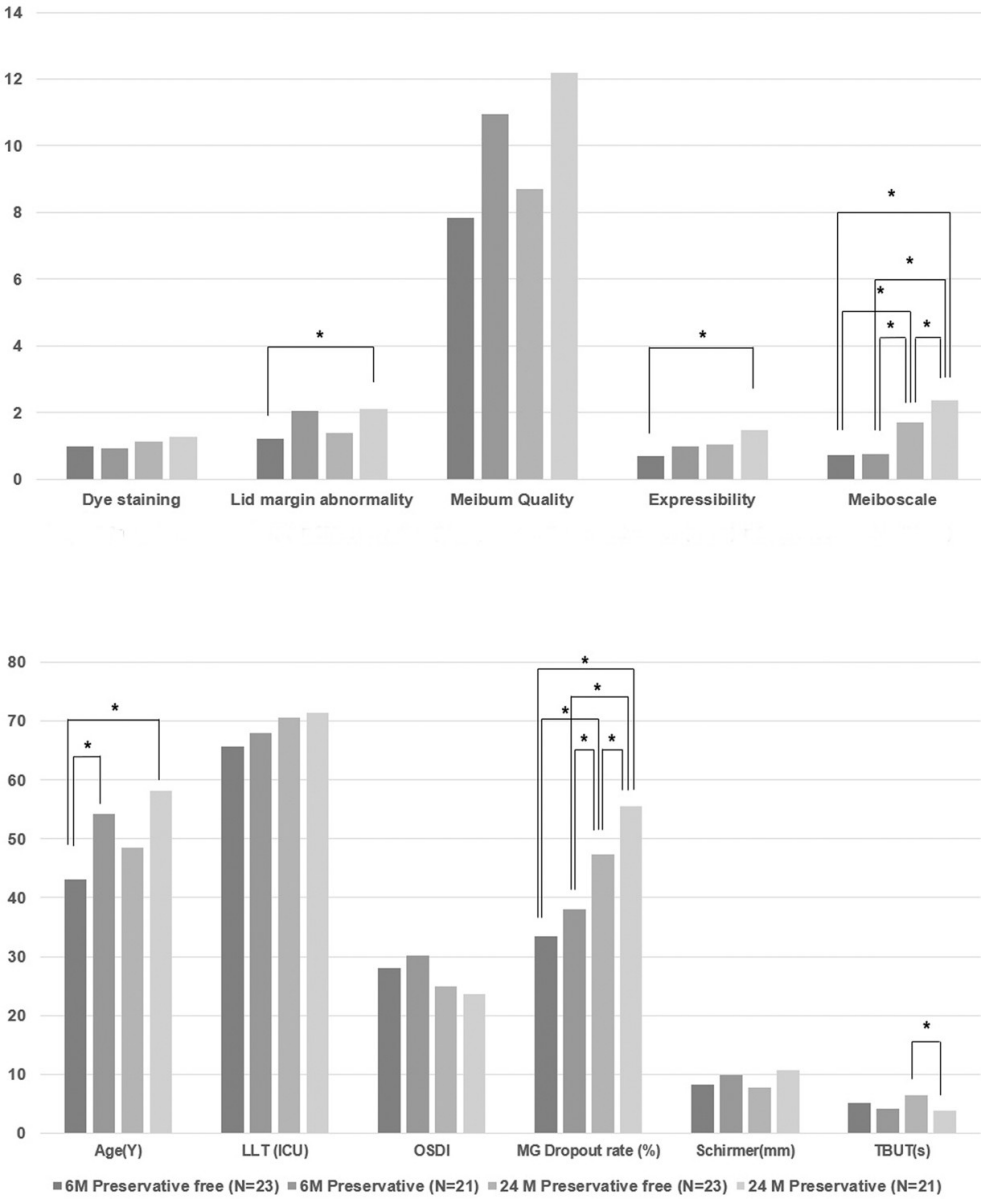
Comparisons of ocular surface and meibomian gland parameters among subgroups. The Tukey method was used as a post hoc analysis after analysis of variance. * indicates significant difference in post hoc analysis. LLT, lipid layer thickness; TBUT, tear breakup time; OSDI, ocular surface disease index; MG, meibomian gland.

We compared age, sex ratio, ocular surface parameters, and MG parameters among four groups: 1) 6-month users of PF-Tafluprost; 2) 6-month users of PC-Tafluprost; 3) 24-month users of PF-Tafluprost; and 4) 24-month users of PC-Tafluprost. There were significant differences in age (p = 0.003), TBUT (p = 0.016), lid margin abnormality (p = 0.016), expressibility (p = 0.039), meiboscale (p<0.001), and MG dropout rate (p<0.001) among the four groups (Table 2). Results of post hoc analyses are shown in Fig 1. There were significant differences in age between 6-month users of PF-Tafluprost and 6-month users of PC-Tafluprost (p = 0.045), as well as between 6-month users of PF-Tafluprost and 24-month users of PC-Tafluprost (p = 0.003). TBUT showed a significant difference only between 24-month users of PF-Tafluprost and 24-month users of PC-Tafluprost (p = 0.022). There were significant differences between 6-month users of PF-Tafluprost and 24-month users of PC-Tafluprost in lid margin abnormality and expressibility (p = 0.048 and p = 0.022, respectively). MG dropout rate and meiboscale showed significant differences in all post hoc analyses, except for comparisons between 6-month users of PF-Tafluprost and 6-month users of PC-Tafluprost.

**Table 2:** Comparison of clinical variables and parameters indicative of ocular surface and meibomian gland condition among study groups using Tafluprost.

	6 M (N = 44)		24 M (N = 44)		
	PF-Tafluprost (N = 23) (Mean ± SD or Ratio)	PC-Tafluprost (N = 21) (Mean ± SD or Ratio)	PF-Tafluprost (N = 23) (Mean ± SD or Ratio)	PC-Tafluprost (N = 21) (Mean ± SD or Ratio)	p*
Age (years)	43.17 ± 11.87	54.24 ± 14	48.43 ± 15.59	58.19 ± 13.24	0.003
Sex (M:F)	11:12	9:12	14:9	11:10	0.668
OD:OS	12:11	16:5	14:9	12:9	0.403
ICU	65.7 ± 23.41	68 ± 22.62	70.61 ± 22.22	71.33 ± 29.43	0.863
TBUT (s)	5.13 ± 2.85	4.19 ± 2.36	6.43 ± 3.94	3.90 ± 1.64	0.018
Dye staining	1 ± 0.97	0.93 ± 1.21	1.13 ± 0.97	1.27 ± 0.83	0.218
Schirmer (mm)	8.35 ± 4.89	9.86 ± 4.08	7.78 ± 5.01	10.76 ± 5.86	0.186
OSDI	27.99 ± 22.26	30.16 ± 23.54	24.96 ± 13.19	23.66 ± 20.35	0.715
Lid margin abnormality	1.22 ± 1.09	2.05 ± 1.28	1.39 ± 0.94	2.10 ± 1.09	0.016
Meibum quality	7.83 ± 5.85	10.95 ± 5.84	8.70 ± 5.87	12.20 ± 5.44	0.053
Expressibility	0.7 ± 0.82	1 ± 1	1.04 ± 1.02	1.48 ± 0.6	0.039
Meiboscale	0.739 ± 0.541	0.762 ± 0.625	1.70 ± 0.82	2.38 ± 0.67	<0.001
MG dropout rate	0.335 ± 0.59	0.380 ± 0.0484	0.473 ± 0.09	0.555 ± 0.1	<0.001

* Analysis of variance or chi-squared test. PF, preservative-free; PC, preservative-containing; LLT, lipid layer thickness; TBUT, tear breakup time; OSDI, ocular surface disease index; MG, meibomian gland

TBUT, lid margin abnormality, expressibility, meiboscale, and MG dropout rate showed significant differences in comparisons among the groups. Correlations among these parameters were identified by multiple regression analyses including age, sex, medication duration, and preservative status (Table 3). TBUT was significantly correlated with preservative status (p = 0.005). Age and expressibility were significantly correlated with lid margin abnormality (p = 0.004 and p = 0.023, respectively). Expressibility was significantly correlated with age (p = 0.003), sex (p = 0.04), lid margin abnormality (p = 0.023), and meiboscale (p<0.001). Three parameters showed significant correlations with MG dropout rate: medication duration, preservative status, and meiboscale (p<0.001, p = 0.024, and p<0.001, respectively). Meiboscale showed significant correlations with medication duration (p = 0.003), expressibility (p = 0.037), and MG dropout rate (p<0.001).

**Table 3:** Correlations between clinical variables and parameters indicative of ocular surface and meibomian gland condition.

		TBUT	Lid margin abnormality	Expressibility	MG Dropout rate	Meiboscale
Age	β	0.02	0.03	0.02	0.001	-0.01
	(95% CI)	(-0.03 to 0.08)	(0.01 to 0.04)	(0.01 to 0.03)	(-0.001 to 0.002)	(-0.02 to 0.01)
	P	0.419	0.004	0.003	0.253	0.447
Sex	β	-0.27	0.17	-0.47	0.001	0.06
(male reference)	(95% CI)	(-1.61 to 1.06)	(-0.26 to 0.6)	(-0.78 to -0.16)	(-0.03 to 0.03)	(-0.2 to 0.32)
	P	0.687	0.43	0.04	0.931	0.654
Duration	β	0.25	-0.34	-0.06	0.08	0.54
(6M reference)	(95% CI)	(-1.67 to 2.17)	(-0.95 to 0.27)	(-0.53 to 0.41)	(0.04 to 0.12)	(0.19 to 0.89)
	P	0.799	0.271	0.793	< .001	0.003
Preservative	β	-2.05	0.36	-0.08	0.04	0.01
(non-preservative reference)	(95% CI)	(-3.48 to -0.62)	(-0.12 to 0.83)	(-0.44 to 0.29)	(0.01 to 0.07)	(-0.29 to 0.3)
	P	0.005	0.14	0.685	0.024	0.964
TBUT	β		0.02	-0.02	0.001	0.01
	(95% CI)		(-0.05 to 0.09)	(-0.08 to 0.03)	(-0.01 to 0.01)	(-0.03 to 0.06)
	P		0.561	0.447	0.895	0.6
Lid margin abnormality	β	0.2		0.19	-0.003	0.09
	(95% CI)	(-0.49 to 0.9)		(0.03 to 0.36)	(-0.02 to 0.01)	(-0.05 to 0.23)
	P	0.561		0.023	0.681	0.19
Expressibility	β	-0.35	0.33		-0.003	0.19
	(95% CI)	(-1.26 to 0.56)	(0.05 to 0.62)		(-0.02 to 0.02)	(0.01 to 0.36)
	P	0.447	0.023		0.767	0.037
MG dropout rate	β	-0.66	-0.65	-0.36		4.26
	(95% CI)	(-10.49 to 9.18)	(-3.81 to 2.5)	(-2.77 to 2.05)		(2.58 to 5.94)
	P	0.895	0.681	0.767		< .001
Meiboscale	β	0.3	0.24	0.06	0.06	
	(95% CI)	(-0.84 to 1.44)	(-0.12 to 0.61)	(0.04 to 0.08)	(0.04 to 0.08)	
	P	0.6	0.188	< .001	< .001	

TBUT, tear breakup time; MG, meibomian gland

MG dropout rate was the only parameter in the multiple regression analyses that was correlated with both medication duration and preservative status. Table 4 shows the results of the multiple regression analyses to investigate correlations between MG dropout rate and other parameters in each subgroup, stratified by mediation duration and preservative status. In the 6-month user group, only preservative status showed a significant correlation with MG dropout rate (p = 0.015). However, in the 24-month user group, meiboscale was the only parameter significantly correlated with MG dropout rate (p<0.001). In the PC-Tafluprost user group, only meiboscale was significantly correlated with MG dropout rate (p<0.001). However, medication duration and meiboscale were significantly correlated with MG dropout rate among users of PF-Tafluprost (p = 0.002 and p = 0.007, respectively).

**Table 4:** Correlations between MG dropout rate and other parameters based on medication duration or preservative status.

	6 M (N = 44)		24 M (N = 44)		PC-Tafluprost (N = 42)		PF-Tafluprost (N = 46)	
	β (95% CI)	P*	β (95% CI)	P*	β (95% CI)	P*	β (95% CI)	P*
Age	< .001	0.709	0.001	0.493	0.001	0.226	< .001	0.613
	(-0.001 to 0.002)		(-0.001 to 0.003)		(-0.001 to 0.003)		(-0.001 to 0.002)	
Sex	0.002	0.901	< .001	0.999	0.03	0.165	-0.02	0.385
(male reference)	(-0.04 to 0.04)		(-0.05 to 0.05)		(-0.01 to 0.08)		(-0.07 to 0.03)	
Duration					0.07	0.076	0.09	0.002
(6M reference)					(-0.01 to 0.14)		(0.03 to 0.14)	
Preservative	0.05	0.015	0.02	0.555				
(non-preservative reference)	(0.01 to 0.09)		(-0.04 to 0.08)					
TBUT	0.01	0.111	-0.003	0.458	0.004	0.435	-0.001	0.834
	(-0.001 to 0.01)		(-0.01 to 0.01)		(-0.01 to 0.02)		(-0.01 to 0.01)	
Lid margin abnormality	-0.001	0.952	0.01	0.939	-0.01	0.551	-0.01	0.678
	(-0.02 to 0.02)		(-0.03 to 0.03)		(-0.03 to 0.02)		(-0.03 to 0.02)	
Expressibility	-0.004	0.95	-0.01	0.782	-0.01	0.615	0.01	0.739
	(-0.003 to 0.01)		(-0.04 to 0.03)		(-0.04 to 0.03)		(-0.03 to 0.04)	
Meiboscale	0.02	0.273	0.08	< .001	0.07	< .001	0.05	0.007
	(-0.02 to 0.05)		(0.04 to 0.11)		(0.03 to 0.11)		(0.01 to 0.08)	

*Multiple regression. PC, preservative-containing; PF, preservative-free; TBUT, tear breakup time; MG, meibomian gland

## Discussion

Because of their ease of use (once per day) and strong IOP-lowering effects, PGAs have been used as a first-choice medication for treatment of glaucoma [22,23]. Therefore, it is important to evaluate the alteration of MG condition associated with PGAs because MG-related disease can affect medication compliance [24, 25]. Recently, PF-PGAs have been developed, which can be used to reduce the occurrence of ocular surface disorder and MGD associated with the preservatives in topical medications. However, because the active ingredient present in PGAs is associated with the onset of MGD, deterioration of MG status is unavoidable during long-term medication use, even when using PF-PGAs. In the present study, among parameters indicative of ocular surface and MG condition, TBUT, lid margin abnormality score, meibum quality score, meiboscale, and MG dropout rate showed at least one significant difference in group comparisons (Fig 1A and 1B). Among these parameters, MG dropout rate and meiboscale showed a stepwise increasing pattern and significant differences in nearly all comparisons between subgroups. This suggests that the methods for evaluating the deterioration of MG morphology can be used as a way of identifying the negative effect of the preservatives included in the PGAs or the negative effect of the duration of PGAs use.

In the present study, MG dropout rate was significantly correlated with medication duration and preservative status, when the data were adjusted for age and sex. Notably, MG dropout rate was significantly correlated with preservative status in 6-month user group; however, there was no significant correlation between preservative status and MG dropout rate in the 24-month user group. Considering the results of another subgroup analysis conducted under a different preservative status in the present study, which showed a significant correlation between MG dropout rate and medication duration only in the PF-Tafluprost user group, MG dropout rate may represent morphologic changes in the MG that are affected by preservative status and medication duration. Meiboscale, another parameter indicative of morphologic changes of the MG, showed correlation results that were similar to those exemplified by MG dropout rate. However, multivariate analyses showed that preservative status was not correlated with meiboscale; thus, the effect of preservative on MG may not be accurately reflected by meiboscale. Several previous studies indicated that MG loss was affected by age and sex [26–28]. However, because we showed that MG dropout rate was correlated with the duration of Tafluprost use and preservative status, regardless of age and sex, the MG dropout rate may be useful for evaluating MGD status in glaucoma patients using PC- or PF-PGAs.

A number of previous studies have reported that PF anti-glaucoma topical medications have advantages in treatment of ocular surface disease [29–32]. Tear film instability, caused by detergent effects, direct toxicity, and allergic reactions, is a known mechanism of preservative-induced ocular surface disease [29,33–35]. In addition, the induction of chronic inflammation by this mechanism increases the occurrence of MGD [36]. However, if the active ingredient induces MGD, the benefit of using a PF medication may be limited. Our findings support this conclusion.

The MG dropout rate and meiboscale among 24-month users of PF-Tafluprost were higher than those among 6-month users of PC-Tafluprost. In addition, the MG dropout rate was significantly correlated with the presence of preservative only in patients who used Tafluprost for 6 months. These results support the possibility that PF-PGAs may exhibit a limited prevention effect on the development and progression of MGD. However, this limited prevention effect is not an indication that PF-PGAs have no clinical use. Rather, it suggests that PF-PGAs should be the primary consideration when using PGAs in glaucoma patients who have already begun to exhibit atrophic changes in the MG. According to our results, MG dropout rate can be used to identify the glaucoma patients who should use PF-PGAs preferentially by measuring objective status of MG. Further studies are needed to define the cut-off value of MG dropout rate for determining whether PF-PGAs are required.

To investigate the effect of topical anti-glaucoma medication on the ocular surface and MG condition, behavior regarding medication use (e.g., instillation frequency, medication duration, and compliance) as well as the type or concentration of active ingredient and preservative, should be well-controlled. However, it is difficult to conduct research in which these conditions are well-controlled in clinical practice. Therefore, specific formulas, such as burden of anti-glaucoma score, were used to assess the effects of different anti-glaucoma medications in a previous study [6]. We conducted the present study by using only Tafluprost among PGAs. In this manner, we were able to compare changes in the ocular surface and MG condition according to preservative status and medication duration, using a similar active ingredient type, concentration, and instillation frequency. Although we could not identify the precise level of compliance for Tafluprost use in individual patients, we could assume that compliance of Tafluprost was not low, given that this medication required instillation once per day, and that these patients used the same medication (without any changes or addition of new medications) because of the stability of IOP. Therefore, the results of our study were conducted in a condition where confounding factors were controlled as much as possible, which contributes to a high degree of reliability. Based on the results of our study, further analyses are needed to confirm the usefulness of the MG dropout rate for assessment of morphologic alterations in the MG when using other types of anti-glaucoma medications (i.e., non-PGAs).

This study has several limitations. First, we could not confirm the longitudinal change of the MG dropout rate because this study used a cross-sectional design. By performing longitudinal follow-up of the same patients included in this study, patterns of change in MG dropout rate might be identified. Second, we could not evaluate primary conditions of MG and ocular surface in included patients, although we excluded patients whose medical records described abnormal MG findings. Lastly, only Korean patients were included in this study. Because the reported MGD prevalence in the Asian population is higher than that in the Western population [37,38], additional studies are needed to characterize the usefulness of MG dropout rate on various ethnicities.

In conclusion, MG dropout rate in patients using Tafluprost was significantly correlated with medication duration and preservative status. Considering the changing pattern and correlation of MG dropout rate with preservative status and medication duration, MG dropout rate could be used to assess MG status in users of PC-Tafluprost or PF-Tafluprost. This result could be applied to users of other PGAs in addition to Tafluprost.

## Supporting information

S1 Dataset Supporting information file. (XLSX)
